# Amedeo Modigliani (1884-1920). Self-Portrait, 1919.

**DOI:** 10.3201/eid0811.021100

**Published:** 2002-11

**Authors:** Polyxeni Potter

**Affiliations:** * Centers for Disease Control and Prevention, Atlanta, Georgia, USA

**Figure:**
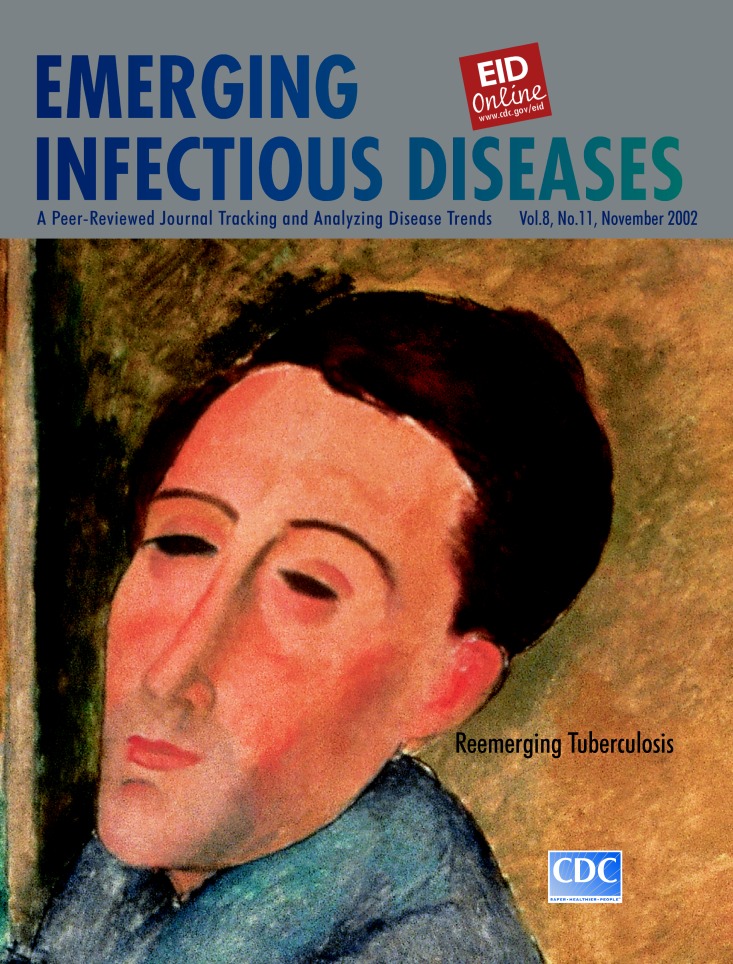
Amedeo Modigliani (1884-1920). Self-Portrait, 1919. Oil on canvas 100 cm x 65 cm. Museu de Arte Contemporanea da Universidade de Sao Paulo, Brazil

Modigliani was born in Livorno, Italy, where he grew up in a Jewish ghetto. He studied art in Florence, and in 1906 he moved to Paris, where he met Pablo Picasso and other leading artists of his era. In Paris, he was influenced by fauvism, the avant-garde art movement promoting a strong, emotional, and nonrealistic use of color, and by his friend the Romanian sculptor Constantin Brancusi, known for his artistic search of pure form. Modigliani was also influenced by African carvings and masks, particularly in his early work, which was mostly sculpture (1).

In his brief life, which even in childhood was marked by ill health, Modigliani was able to grow as an artist and attain his own distinctive style. He is known for his graceful, simplified, and sympathetic portrayal of the human form. His paintings, mostly portraits and studies of the human figure, are characterized by fine sinuous lines and have a simple, spare, and flat appearance, which gives them an almost classical effect. The figures are elongated, the faces oval, and the shapes ethereal, reminiscent at times of Sandro Botticelli (see cover Vol.7, No.3, Emerging Infectious Diseases). The portraits (more than 200 from 1916 to 1919), unburdened by detail, rely on color and shape for emotional and psychological insight and emit a “curious sense of pathos” (1).

Modigliani’s fauvist contemporaries had moved away from the conventional and sentimental in art. They were not interested in the representation of observed reality or even in passion mirrored on a face. They were after “radical simplicity,” the “genius of omission.” Expression to them was achieved through form and spatial depth, the arrangement of line and color on a flat plane and the empty spaces around them (2). In this artistic climate, Modigliani would not have been interested in tuberculosis as a subject for his art, nor would he have painted a conventional portrait of this disease that consumed his adult life and eventually killed him at age 36.

The famous self-portrait on this cover of Emerging Infectious Diseases, painted in 1919, just one year before Modigliani’s death, inspired writers who were captivated by its romanticism to speculate broadly about its meaning. Even though many of the interpretations are mostly conjecture, the length and thinness of the face, as well as its pallor and eerie calmness, may well be due to tuberculosis (3).

In this portrait, so reminiscent of the African masks that had fascinated him not for their intense expression but for their formal simplicity and coherence, Modigliani seems to have captured the essence of his subject, himself. He turned to his fauvist roots for the striking hues so typical of tuberculous complexion, to his friend Brancusi’s sculptures for the studied serenity, and to his own emotional capacity for the depths of darkness welled in those stylized eyes. But whether he intended it or not, the master portrait painter, Modigliani, in this self-portrait of hollowed cheeks and sealed lips, painted more than his face. He painted the face of tuberculosis.

## References

[1] Amedeo Modigliani—Self portrait: famous art reproductions 2002 [cited 2002 September 17]. Available from URL: http://www.famousartreproductions.com/modiglianibiography.html

[2] Janson HW, Janson AF. History of art. New York: Harry N. Abrams, Inc.; 2001.

[3] Chretien J. Tuberculosis: the illustrated history of a disease. Hauts-de-France; 1998.

